# The effects of time-restricted feeding on early vascular, liver, and renal structural changes, oxidative stress, and inflammation in obese rats

**DOI:** 10.1038/s41598-025-20502-y

**Published:** 2025-10-15

**Authors:** Ahmad Khusairi Azemi, Siti Safiah Mokhtar, Anani Aila Mat Zin, Susan Wai Sum Leung, Aida Hanum Ghulam Rasool

**Affiliations:** 1 Institute of Climate Adaptation and Marine Biotechnology, Universiti Malaysia Terengganu, Kuala Nerus, 21030 Terengganu Malaysia; 2 Department of Pharmacology, School of Medical Sciences, Universiti Sains Malaysia, Health Campus, Kota Bharu, 16150 Kelantan Malaysia; 3 Hospital Universiti Sains Malaysia, 16150 Kota Bharu, Kubang Kerian, Kelantan Malaysia; 4 Department of Pathology, School of Medical Sciences, Universiti Sains Malaysia, Kota Bharu, 16150 Kelantan Malaysia; 5 Department of Pharmacology and Pharmacy, Li Ka Shing Faculty of Medicine, The University of Hong Kong, Pok Fu Lam, Hong Kong China

**Keywords:** Obesity, Time-restriction feeding, Intermittent fasting, Oxidative stress, Inflammation, Atherogenic index, Neurological disorders, Nutrition disorders, Cardiovascular diseases, Vascular diseases

## Abstract

Cardiovascular disease remains a leading global cause of death, highlighting the need for new strategies to improve cardiovascular health. Time-restricted feeding (TRF), which limits daily food intake to a specific window, has shown promise in improving metabolic health and supporting weight control. This study investigated the effects of TRF in an obese rat model induced by a high-fat diet (HFD), focusing on early vascular, liver, and kidney structural changes, as well as oxidative stress and inflammation. Thirty male Sprague Dawley rats were assigned to five groups: a normal diet group (NOR), a normal chow with TRF (NOR + TRFNC), a continued HFD group (OB), an HFD with TRF group (OB + TRFHFD), and a group switched to TRF with normal chow (OB + TRFNC). Obesity was induced in three groups over six weeks, followed by a six-week intervention phase. TRF involved fasting for 16 h daily (5:00 p.m. to 9:00 a.m.). TRF led to improved lipid profiles and atherogenic indices in obese rats, regardless of diet. Elevated liver enzymes, alanine aminotransferase (ALT), aspartate aminotransferase (AST), and alkaline phosphatase (ALP) in obese rats were normalized by TRF. Additionally, TRF increased vascular superoxide dismutase (SOD) and decreased malondialdehyde (MDA), interleukin-6 (IL-6), and tumor necrosis factor-alpha (TNF-α). Histological analysis showed that fat infiltration and steatosis in the liver were reduced by TRF. Renal and vascular structures also showed improvement. In conclusion, TRF exhibits anti-atherosclerotic effects, likely due to reduced vascular oxidative stress, inflammation, improved liver and kidney function, and better atherogenic profiles. These benefits were supported by histopathological findings in hepatic and renal tissues.

Cardiovascular disease remains the leading cause of premature mortality worldwide, responsible for over 17.3 million deaths annually. By 2030, this number is projected to exceed 23.6 million, with over three-quarters of these deaths occurring in low- and middle-income countries. Diet is a significant contributor to cardiovascular disease, linked to approximately 7.94 million deaths each year^1^.

Obesity, a major risk factor for type 2 diabetes mellitus (T2DM) and cardiovascular disease, is a key component of metabolic syndrome (MetS), a condition characterized by insulin resistance, high triglycerides, low high-density lipoprotein-cholesterol (HDL-C) levels, and hypertension^2–4^. MetS affects over 25% of the global population^5^. Managing MetS typically involves dietary changes, increased physical activity, and therapeutic interventions to restore optimal levels of triglycerides (TG), low-density lipoprotein-cholesterol (LDL-C), HDL-C, blood glucose, and blood pressure^6,7^.

Prevention strategies, particularly dietary modifications, are crucial in reducing cardiovascular disease risk. Intermittent fasting, characterized by alternating periods of eating and fasting, has gained attention for its potential benefits in weight loss and body composition improvement^3,8,9^. Among these protocols, time-restricted feeding (TRF), which limits the daily eating window to 4 to 12 h (h), shows promise, although prospective studies on its benefits are limited^10^. TRF is recognized for its potential to improve insulin sensitivity, blood pressure, and oxidative stress, even without weight loss^3,11,12^. However, the relationship between weight loss and changes in traditional blood biomarkers, such as glycemia and lipid profiles, remains inconsistent^3^. Given the complexity of obesity, these biomarkers should be considered alongside body composition when evaluating weight loss progress.

TRF supports circadian rhythm synchronization by maintaining a consistent daily cycle of feeding and fasting^13^. Disruptions to these rhythms, caused by erratic eating patterns or extended eating periods, can increase the risk of MetS components, including obesity, hypertension, insulin resistance, inflammation, and dyslipidemia^13–15^. Observational studies in humans and animal models suggest that maintaining a daily rhythm of feeding and fasting can prevent and reverse metabolic diseases^16–18^.

Emerging evidence suggests that TRF, which extends nighttime fasting to over 12 h, may improve key cardiovascular health indicators^19^. In animal models, restricting feeding to their active phase has shown benefits in metabolic health and obesity prevention^20,21^. Human studies also link TRF with reduced body weight, blood pressure, and inflammation, though findings are not always consistent^22–25^. To date, no studies have directly explored the association between nighttime fasting duration and cardiovascular disease risk, emphasizing the need for further investigation into the impact of meal timing and nighttime fasting on cardiometabolic health. Therefore, in this study, we hypothesized that TRF for six weeks in a HFD-induced obese rat model would attenuate MetS, oxidative stress, inflammation, and early vascular, liver, and renal structural changes.

## Results

### Body weight, levels of serum lipid profiles, atherogenic index, and liver function test

**Fig. 1:**
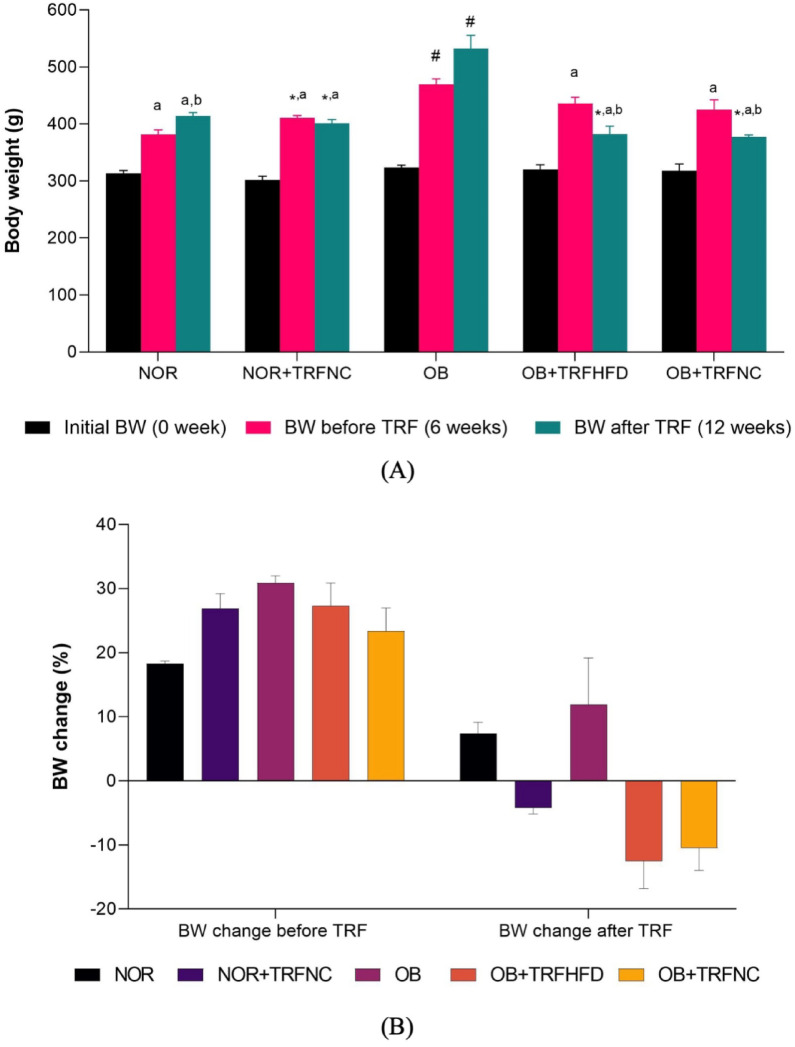
Body weight of the normal and obese rats with TRF. **A** Initial body weight, body weight before, and after TRF. **B** Body weight change before TRF and after TRF. Data are expressed as mean ± SEM (*n* = 6). ^#^*p* < 0.05 vs. NOR group; *p < 0.05, vs. OB group. ^a^*p* < 0.05 vs. initial BW within the group; ^b^*p* < 0.05 vs. BW before TRF within the group.

The body weight of rats in the five groups is shown in Fig. 1. In the current study, there are no significant differences in the initial body weight among the study groups. After six weeks of HFD feeding, all the groups showed weight gains compared to their initial body weight. The final body weight (week 12) in the OB group was significantly higher compared to the NOR group. TRF administration with normal chow and HFD in the obese groups significantly reduced body weight compared to the OB group. This effect was in line with the administration of TRF in the normal group (NOR + TRFNC). The percentage changes of body weight before and after TRF have been illustrated in Fig. 1B.

**Fig. 2:**
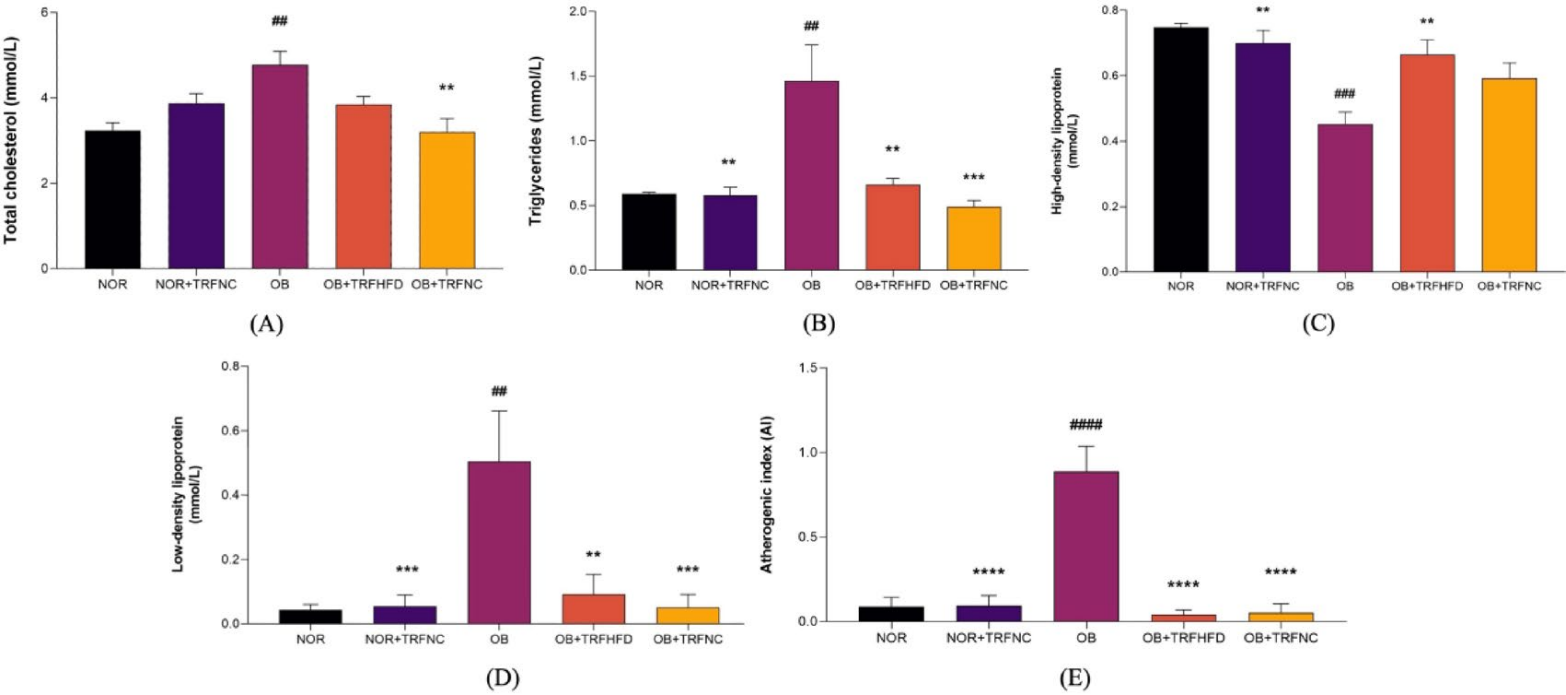
Levels of serum lipid profiles (**A**-**D**) and atherogenic index (**E**) of the normal and obese rats with TRF. Data are expressed as mean ± SEM (*n* = 6). ^##^*p* < 0.01, ^###^*p* < 0.001, ^####^*p* < 0.0001 vs. NOR group; **p < 0.01, ***p < 0.001, ****p < 0.0001 vs. OB group.

In this study, the OB group exhibited significantly elevated levels of total cholesterol (TC, 32.3%), TG (59.6%), LDL-C (91.3%), and atherogenic index (AI, 89.9%) compared to the NOR group (*p* < 0.05) (Fig. 2). Conversely, HDL-C (65.4%) levels were markedly reduced in the OB group relative to NOR. Administration of TRF alongside a HFD for six weeks in obese rats resulted in a significant decrease in TC (19.4%), TG (54.7%), LDL-C (81.8%), and AI (95.4%) levels compared to the untreated obese group. Furthermore, obese rats fed a normal chow diet with TRF for six weeks showed a significant reduction in TC (33%), TG (66.4%), LDL-C (89.8%), and AI (94.2%) levels compared to the OB group. The NOR + TRFNC group also demonstrated significant reductions in TG, LDL-C, and AI, along with a notable increase in HDL-C, when compared to the obese controls.

**Fig. 3:**
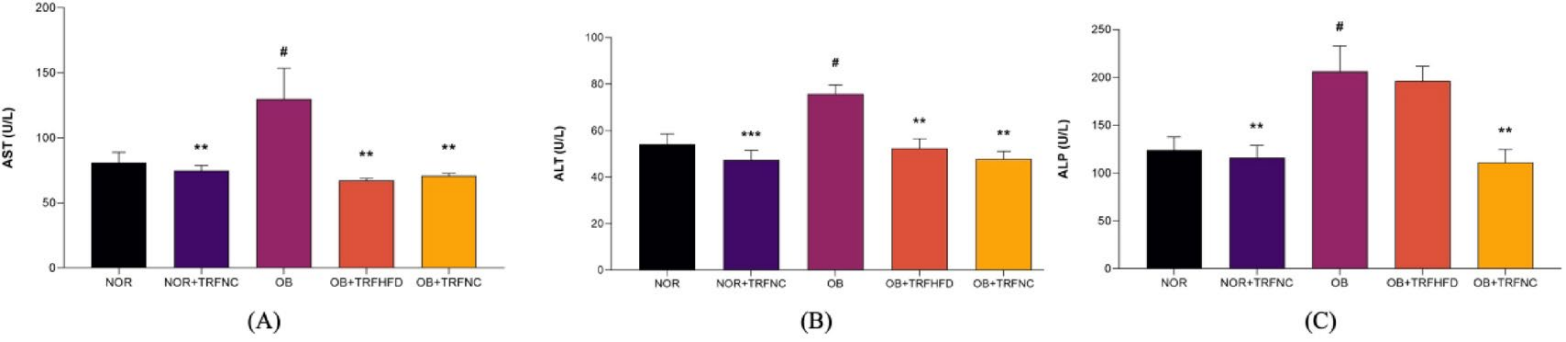
Levels of liver function test for AST (**A**), ALT (**B**), and ALP (**C**) of the normal and obese rats with TRF. Data are expressed as mean ± SEM (*n* = 6). ^#^*p* < 0.05 vs. NOR group; **p < 0.01, ***p < 0.001 vs. OB group.

In the present study, the levels of AST (60.2%), ALT (39.8%), and ALP (66.6%) were significantly (*p* < 0.05) higher in the OB group compared to the NOR group. TRF with HFD for six weeks significantly reduced AST (48.3%) and ALT (30.8%) levels in obese rats compared to the OB group. TRF with a normal chow in obese rats for six weeks significantly reduced AST (45.7%), ALT (36.9%), and ALP (46.2%) compared to the OB group (Fig. 3). Administration of TRF with a normal chow in normal rats significantly reduced AST, ALT, and ALP compared to the OB group.

### Aortic tissue MDA and SOD levels

**Fig. 4:**
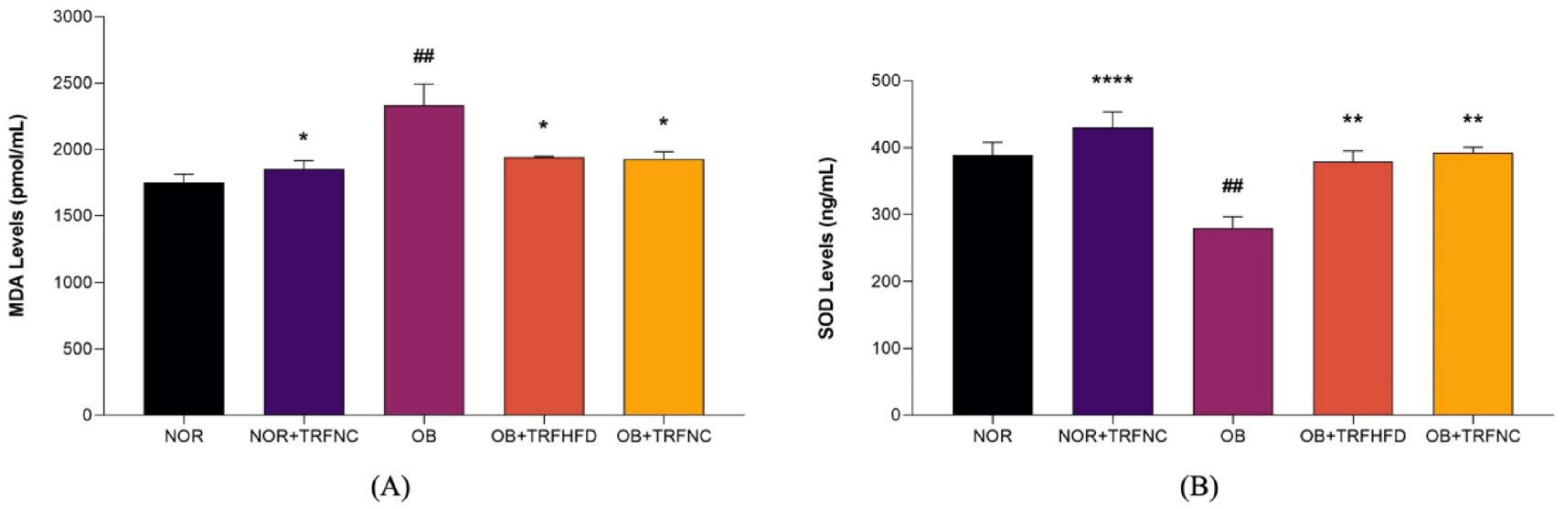
Effect of TRF on aortic tissue MDA (**A**) and SOD (**B**) levels in the normal and obese groups. Data are presented as mean ± SEM (*n* = 6). ^##^*p* < 0.01 vs. NOR group; *p < 0.05, **p < 0.01, ****p < 0.0001 vs. OB group.

In the present study, twelve weeks of HFD feeding in the OB group significantly increased aortic MDA levels (NOR: 1750 ± 63.19 vs. OB: 2332 ± 160.50 pmol/mL, 25.0%; *p* = 0.0023) and lower SOD levels (NOR: 388.20 ± 19.42 vs. OB: 279.60 ± 16.86 ng/mL, 28.0%; *p* = 0.0042) compared to NOR groups (Fig. 4). TRF with HFD (OB + TRFHFD: 1943 ± 4.08 vs. OB: 2332 ± 160.50 pmol/mL, 16.7%; *p* = 0.0363) and normal chow (OB + TRFNC: 1928 vs. OB: 2332 ± 160.50 pmol/mL, 17.3%; *p* = 0.0283) significantly reduced aortic MDA levels in obese rat model levels compared to OB group (Fig. 4A). In addition, TRF with a normal chow (NOR + TRFNC: 1852 ± 63.68 vs. OB: 2332 ± 160.50 pmol/mL, 20.6%; *p* = 0.0123) in normal rats significantly reduced aortic MDA levels compared to OB group.

The vascular tissue SOD levels were increased in TRF with HFD (OB + TRFNC: 379.30 ± 15.56 vs. OB: 279.60 ± 16.86 ng/mL, 26.3%; *p* = 0.0054) and normal chow (OB + TRFNC: 392.40 ± 7.76 vs. 279.60 ± 16.86 ng/mL, 28.7%; *p* = 0.0030) in obese rat model compared to OB group (Fig. 4B). In addition, TRF with a normal chow (NOR + TRFNC: 430.40 ± 23.34 vs. OB: 279.60 ± 16.86 ng/mL, 35.0%; *p* < 0.0001) in normal rats significantly increased aortic SOD levels compared to OB group.

## Aortic tissue TNF-α and IL-6 levels

**Fig. 5:**
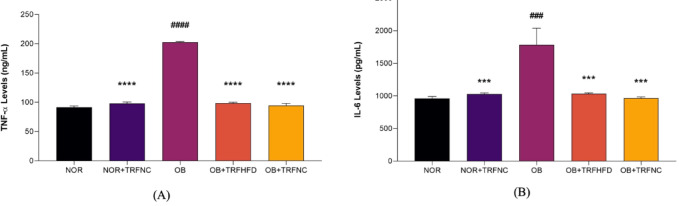
Effect of TRF on aortic tissue TNF-α (**A**) and IL-6 (**B**) levels in the normal and obese groups. Data are presented as mean ± SEM (*n* = 6). ^###^*p* < 0.001, ^####^*p* < 0.0001 vs. NOR group; ***p < 0.001, ****p < 0.0001 vs. OB group.

In the present study, twelve weeks of HFD in the OB group significantly increased aortic TNF-α (NOR: 91.52 ± 2.25 vs. OB: 202.60 ± 1.24 ng/mL, 54.8%; *p* < 0.0001) and IL-6 (NOR: 960.00 ± 31.69 vs. OB: 1784.00 ± 254.80 pg/mL, 46.2%; *p* = 0.0004) levels compared to NOR groups (Fig. 5). TRF with HFD (OB + TRFHFD: 98.43 ± 1.57 vs. OB: 202.60 ± 1.24 ng/mL, 51.4%; *p* < 0.0001) and normal chow (OB + TRFNC: 94.57 ± 3.54 vs. OB: 202.60 ± 1.24 ng/mL, 53.3%; *p* < 0.0001) significantly reduced aortic TNF-α levels in obese rat model levels compared to OB group (Fig. 5A). In addition, TRF with a normal chow (NOR + TRFNC: 97.97 ± 2.52 vs. OB: 202.60 ± 1.24 ng/mL, 51.6%; *p* < 0.0001) in normal rats significantly reduced aortic TNF-α levels compared to OB group.

The vascular tissue IL-6 levels were reduced in TRF with HFD (OB + TRFHFD: 1033.00 ± 14.63 vs. OB: 1784.00 ± 254.80 pg/mL, 42.1%; *p* = 0.0007) and normal chow (OB + TRFNC: 966.30 ± 17.84 vs. OB: 1784.00 ± 254.80 pg/mL, 45.8%; *p* = 0.0005) in obese rat model compared to OB group (Fig. 5B). In addition, TRF with a normal chow (NOR + TRFNC: 1027.00 ± 19.66 vs. OB: 1784.00 ± 254.80 pg/mL, 42.4%; *p* = 0.0006) in normal rats significantly reduced aortic IL-6 levels compared to OB group.

## Histological changes in the thoracic aorta, liver, and kidney

### Thoracic aorta and aortic intima-media thickness

**Fig. 6:**
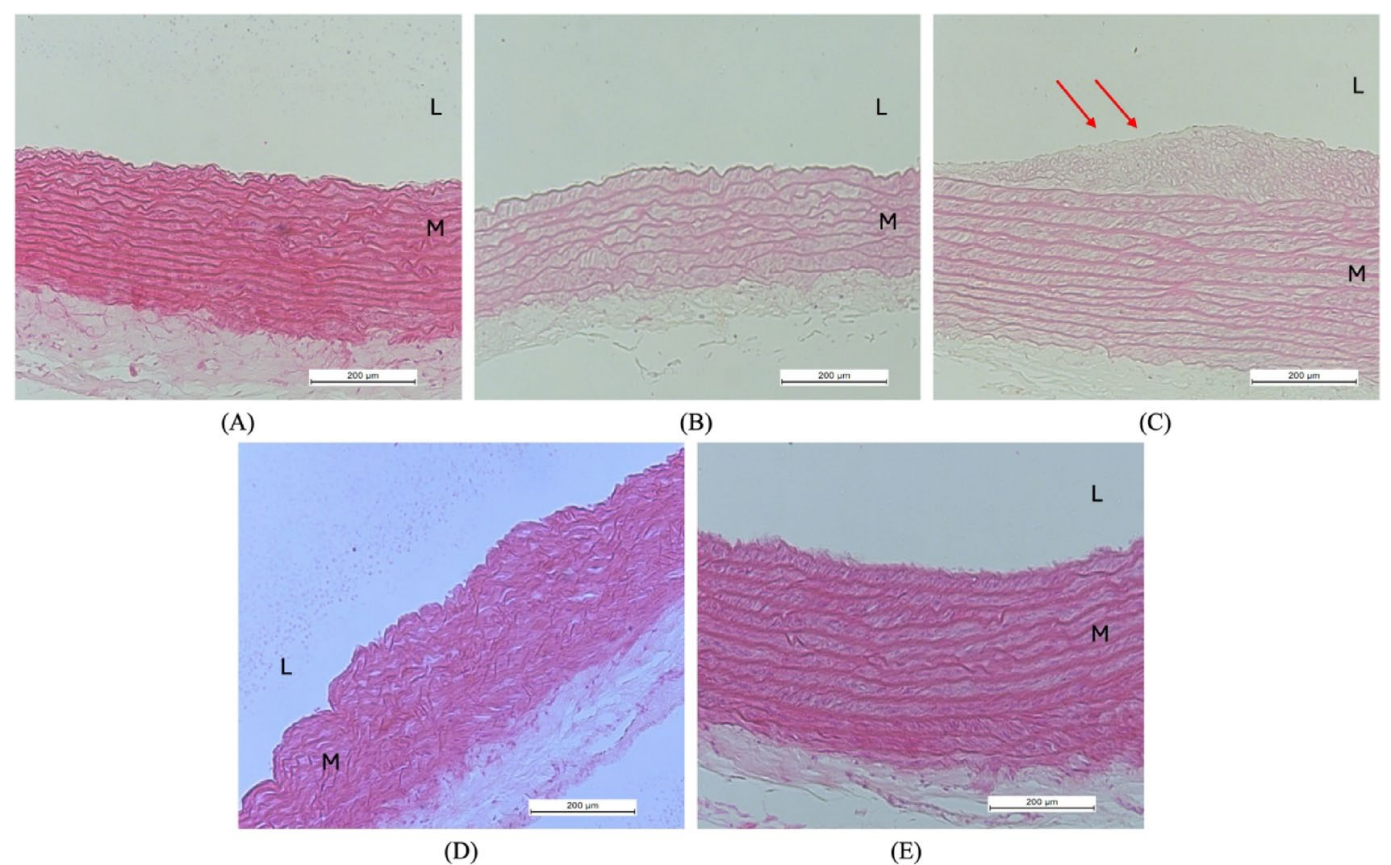
Histopathological changes in thoracic aortas of rats after 6 weeks of TRF (magnification, H&E ×200). (**A**) The non-obese control group (NOR) showed an intact vascular layer and no impairment of the vessel wall. (**B**) The non-obese group with TRF (NOR + TRFNC) showed an intact vascular layer and no impairment to vessel integrity. (**C**) In the obese control group (OB), the aortas appeared to be thick and exhibited disorientation of the smooth muscle cells with foam cell formation. Red arrows indicate foam cells (**D**). In the obese group with TRF fed with HFD (OB + TRFHFD), the aortas showed a thinner vascular wall compared to the OB aortas, no impairment of the vascular wall, and no foam cell formation. (E) In the obese group with TRF fed with a normal chow (OB + TRFNC), the aortas showed a thinner vascular wall compared to the OB aortas, no impairment of the vascular wall, and no foam cell formation. L: vascular lumen; M: tunica media layer.

**Fig. 7:**
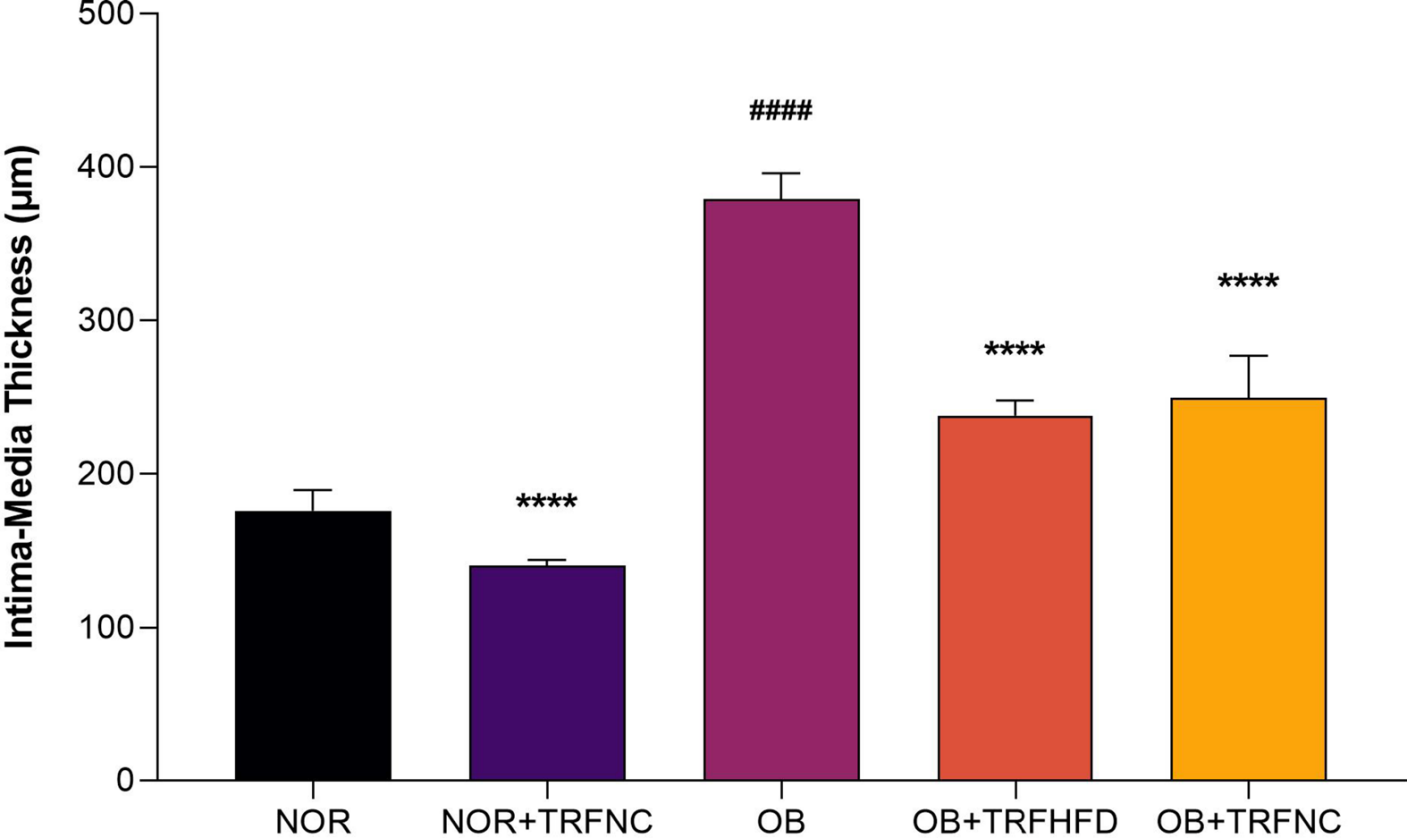
IMT of the thoracic aorta in the non-obese and obese rats with TRF. The obesity groups with TRF had reduced aortic IMT compared to the obese control group (OB). Data presented as mean ± SEM (*n* = 6). ^####^*p* < 0.0001 vs. NOR group; ****p < 0.0001, vs. OB group.

Figure 6 presents hematoxylin and eosin (H&E) stained histological sections of the thoracic aorta. The vascular morphology in both the NOR and NOR + TRFNC groups appeared normal, with well-preserved structural layers and no signs of vascular damage (Fig. 6A and B). In contrast, the aortic tissue from the OB group displayed increased wall thickness, disorganized smooth muscle cells, and the presence of foam cells (Fig. 6C). However, in the OB + TRFHFD and OB + TRFNC groups, the aortic sections showed improved vascular structure, with relatively thinner walls and no evidence of foam cell accumulation or disruption of vessel integrity (Fig. 6D and E). The thickness of the intima and media layers, the two inner layers of the thoracic aorta, was measured to obtain the thoracic intima-media thickness (IMT) (Fig. 7). The aortic IMT in the OB group was 2.16 times thicker compared to the NOR group (*p* < 0.0001). In non-obese rats, TRF combined with a standard chow diet resulted in significantly reduced aortic IMT compared to the obese control group (*p* < 0.0001). However, there was no notable difference in aortic IMT between the NOR and the group receiving both normal chow and TRF (NOR + TRFNC). Similarly, the obese rats subjected to TRF with a high-fat diet (OB + TRFHFD) exhibited a 37% reduction in IMT compared to the obese controls (*p* < 0.0001). Additionally, the OB + TRFNC group showed a 34% lower IMT relative to the OB group (*p* < 0.0001). No statistically significant difference in IMT was observed between the OB + TRFNC and OB + TRFHFD groups (*p* = 0.9851).

## Liver

**Fig. 8:**
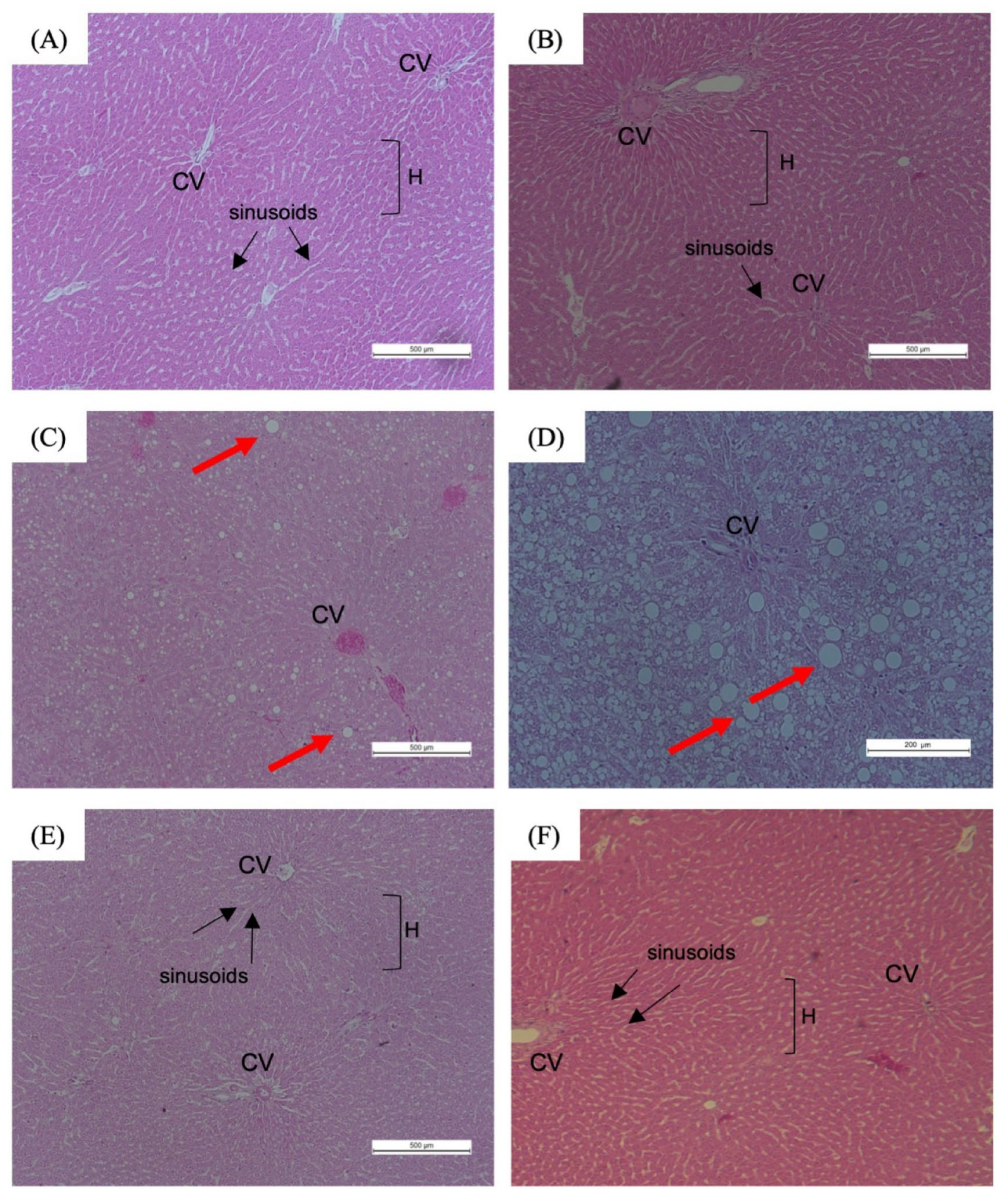
Histopathological observation of liver tissues of non-obese and obese rats with TRF at H&E ×100 and ×200 magnification. The tissue section of NOR **A** and NOR + TRFNC **B** groups demonstrated normal liver architecture with normal hepatic cells. **C**, **D** The hepatic liver tissue of the OB group showed features of hepatic steatosis with numerous large and small lipid droplets visible through the hepatocytes. Red arrows (➙) showed the lipid droplets. Hepatic tissue of OB + TRFHFD **E** and OB + TRFNC **F** groups demonstrated normal liver tissue architecture with the presence of normal hepatic cells. CV: central vein; H: hepatocytes.

Figure 8 illustrates the microscopic examination of liver tissues from obese rats on a HFD following TRF. Liver histology from both NOR and NOR + TRFNC revealed typical hepatic structure, characterized by intact hepatocytes, clearly defined nuclei, preserved cytoplasm, distinct sinusoidal spaces, and largely visible central veins (Fig. 8A and B). In contrast, liver sections from the OB group showed pathological alterations consistent with fatty liver changes. Numerous large and small lipid droplets (clear, round vacuoles) are visible throughout the hepatocytes, indicating significant fat accumulation (Fig. 8C and D). This disruption in normal hepatocyte architecture suggests metabolic stress, likely due to a HFD. Additionally, there are no prominent signs of inflammatory cell infiltration or fibrosis at this magnification. These findings align with hepatic steatosis, commonly observed in obesity models. OB + TRFHFD and OB + TRFNC demonstrated mild lipid accumulation but showed a normal appearance of liver architecture, indicated by the presence of normal hepatic cells with the characteristic morphology of well-preserved cytoplasm, prominent nucleus, single-layer spaces, and unremarkable portal triad (Fig. 8E and F). Therefore, TRF administration reduced the architectural damage to the liver. Table 1 showed the liver lesion scoring in the non-obese and obese rats with TRF. From Table 1, it was shown that OB rats showed an early stage of steatosis with the presence of lipid droplets (Fig. 8C and D). Other than that, other groups showed a normal architecture of the liver without any changes.

**Table 1:** The rat’s liver lesion scoring of non-obese and obese rats with TRF.

	NOR	NOR+ TRFNC	OB	OB+ TRFHFD	OB+ TRFNC
Steatosis					
Grade	0	0	1	0	0
Location	0	0	1	0	0
Microvesicular	0	0	1	0	0
Inflammation					
Microgranulomas	0	0	0	0	0
Lipogranulomas	0	0	0	0	0
Portal inflammation	0	0	0	0	0
Cellular features					
Hepatocytes hypertrophy/ Balloning	0	0	1	0	0
Acidophilic bodies	0	0	0	0	0
Pigmented macrophages	0	0	0	0	0
Megamitochondria	0	0	0	0	0
Mallory’s hyaline	0	0	0	0	0
Glycogenated nuclei	0	0	0	0	0
NAS score	0	0	4	0	0
Fibrosis score	0	0	0	0	0

NAS: NAFLD activity score; NAFLD: non-alcoholic fatty liver disease.

### Kidney

**Fig. 9:**
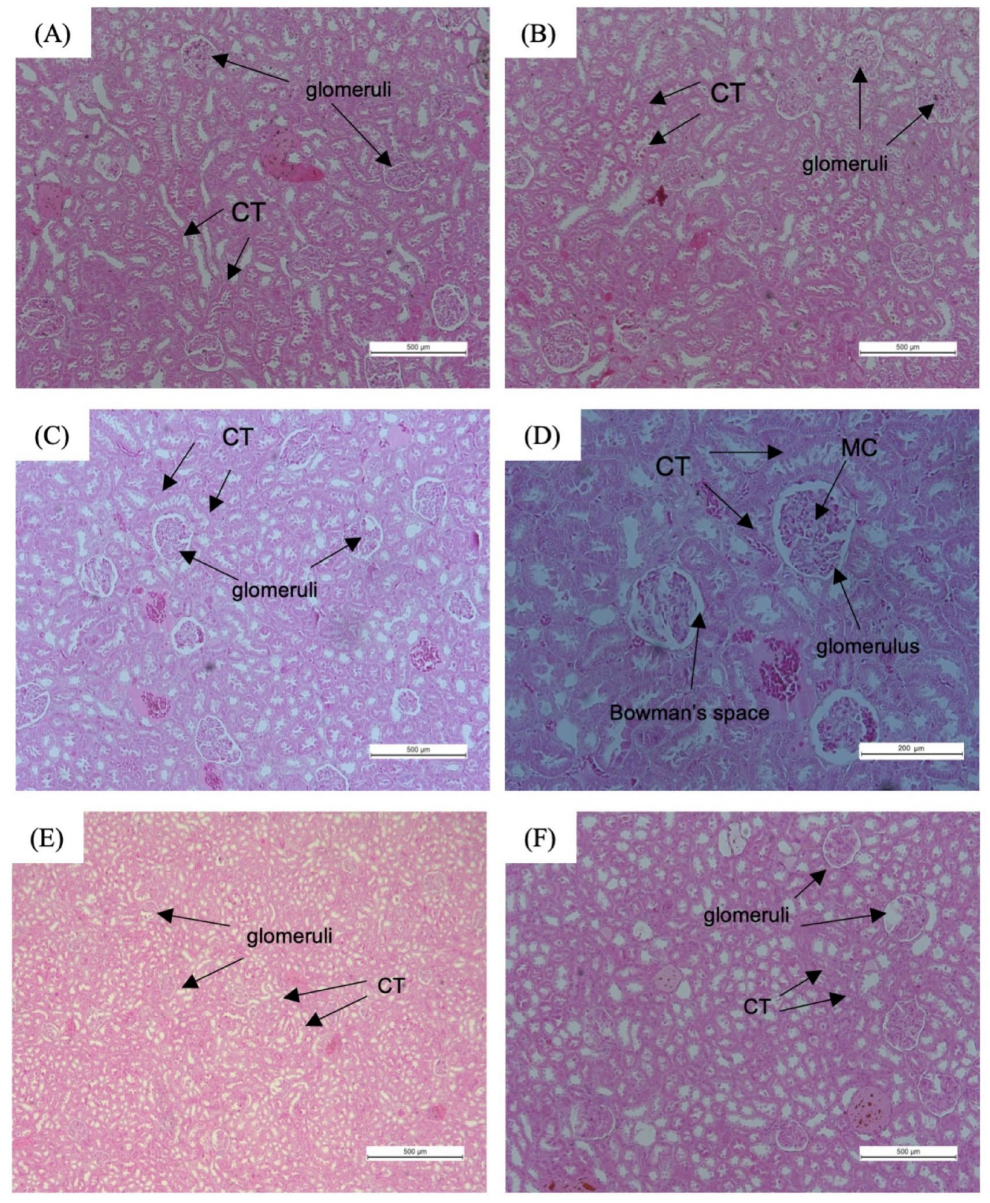
Histopathological observation of renal tissues of non-obese and obese rats with TRF at magnifications ×100 and ×200. Renal tissue sections of NOR (**A**) and NOR + TRFNC (**B**) showed normal architecture. **C**, **D** The renal tissue structure of the OB group showed signs of glomerular congestion, tubular injury, and interstitial changes. Renal tissue of OB + TRFHFD **E** and OB + TRFNC **F** groups appears normal in architecture, and the glomerular are relatively preserved. CT: convoluted tubule; MC: mesangial cell.

Figure 9 shows the histopathological findings of renal tissues of non-obese and obese rats. Renal tissue sections of the NOR and NOR + TRFNC groups showed the normal architecture of renal tissue, and the glomeruli appear relatively preserved (Fig. 9A and B). However, in the OB group, the renal tissue shows signs of glomerular congestion, tubular injury, and interstitial changes (Fig. 9C and D), which may be linked to obesity and hyperlipidemia. There is an increase in cellularity, and mild shrinkage is observed in several glomeruli, indicating the onset of early glomerular damage. Obese rats subjected to TRF with HFD and normal chow display relatively intact renal tissue architecture; the glomeruli appear mostly preserved, though some show mild mesangial expansion and thickening of glomerular capillary walls (Fig. 9E and F).

## Discussion

Hyperlipidemia, vascular oxidative stress, and inflammation play key roles in the initiation and progression of atherosclerosis^26,27^. In this study, an obese rat model exhibited significant hyperlipidemia, along with elevated AI levels, vascular oxidative stress, and inflammation. These were associated with thick IMT, with the presence of foam cells in the aortic tissue of obese rats. These parameters in an obese animal model (hyperlipidemia, high AI, increased oxidative stress, and inflammation) provide a suitable model for evaluating the anti-atherosclerotic effects of TRF. The findings in the current study demonstrated that TRF administration in obese rats significantly reduced body weight, TC, TG, LDL-C, and AI levels, while also lowering serum AST, ALP, and ALT compared to obese rats not on TRF (OB). Additionally, TRF increased HDL-C levels, decreased vascular MDA, TNF-α, and IL-6 levels, and elevated SOD levels in the aortic tissue of TRF-treated obese rats. Furthermore, obese rats on TRF exhibited lower aortic IMT values than OB rats. This was associated with an absence of foam cells in the aortic tissue of OB rats with TRF. Other than that, administration of TRF in normal rats showed comparable effects in all parameters compared to the control normal rats. The present study highlights early structural and biochemical alterations in the liver, kidney, and vascular tissues, alongside vascular oxidative stress and inflammation markers. We show that TRF not only improves lipid and atherogenic indices but also reduces vascular oxidative stress and lowers pro-inflammatory cytokines, normalizes liver enzymes, and alleviates hepatic steatosis and renal alterations. Histological findings further substantiate these protective effects. Thus, the novelty of the current work lies in its comprehensive evaluation of multi-organ protective effects of TRF, moving beyond vascular signaling mechanisms^4^ to reveal systemic benefits against oxidative stress, inflammation, and tissue injury. Together, our current and previous^4^ reports complement each other, with the first establishing mechanistic vascular improvements and the present study demonstrating broader organ-level protection, thereby reinforcing the therapeutic potential of TRF in obesity-induced complications.

The AI levels, defined by the ratio of LDL-C to HDL-C, serve as a reliable indicator for early-stage atherosclerosis. In this study, OB rats exhibited higher AI values compared to their normal counterparts (NOR), suggesting increased cardiovascular risk. Elevated AI has also been reported in experimental models of diabetes^26,28,29^ and atherosclerosis^30,31^. LDL-C, commonly labeled as “bad cholesterol,” plays a central role in lipid imbalance and plaque formation. Under conditions of oxidative stress, often associated with hyperlipidemia, LDL-C is converted to its oxidized form (oxLDL-C), which promotes endothelial injury and facilitates the development of atherosclerotic lesions. As such, strategies aimed at lowering LDL-C and preventing its oxidation are crucial for reducing atherosclerotic risk. In this investigation, rats in the OB + TRFHFD group showed marked improvements, including reductions in TC by 19.4%, TG by 54.7%, and LDL-C by 81.8%, along with a 46.9% increase in HDL-C levels, compared to the untreated obese group. These results align with earlier studies that demonstrated the lipid-lowering benefits of TRF in obese animal models^32,33^.

Obesity-associated hyperlipidemia causes excessive reactive oxygen species production, which can lead to an increase in inflammatory mediator expression and the subsequent progression of atherosclerosis^34–36^. In the present study, higher MDA levels and lower SOD levels in the aortas of obese rats demonstrated the presence of oxidative damage and impaired antioxidant capacity. Increased aortic oxidative stress leads to further damage to vascular tissues, which plays a significant role in the development of atherosclerosis^37,38^. Interestingly, this current study demonstrated that TRF administration reduced MDA levels, as well as increased SOD levels in the thoracic aortas of obese rats fed with HFD. The effects of TRF in reducing MDA levels and increasing SOD levels were comparable to those in obese rats fed with normal chow. The findings in the current study were in line with previous findings reported in animal^39–41^ and human^42^ models. A study by Suhaimi et al.^39^ has demonstrated that administration of TRF in an obese prediabetic rat model reduced aortic MDA and increased aortic SOD levels compared to the obese control rat. In addition, studies by Arabmoazzen et al.^41^ and Xiong et al.^40^ have shown that intermittent fasting administration significantly reduced MDA levels in diabetic animal models.

Inflammation is a key mediator of endothelial dysfunction and plays a crucial role in the development of atherosclerosis. Hyperlipidemia and obesity exacerbate this process by inducing chronic low-grade inflammation and oxidative stress, thereby creating a pro-atherogenic environment^27,34^. Within the intimal layer of the arterial wall, activated monocytes infiltrate the endothelium and secrete various inflammatory cytokines, particularly TNF-α, which contribute to endothelial cell damage, enhance leukocyte adhesion, and facilitate the progression of atheromatous plaque formation^26,43,44^. In the present study, administration of TRF to obese rats fed either a HFD or a normal chow significantly reduced aortic TNF-α levels by 51.4% and 53.3%, respectively, compared to obese control rats. The effect of TRF on TNF-α levels in obese rats was comparable to the TNF-α values in non-obese rats with TRF. Interestingly, this reduction in aortic TNF-α levels in the obese rat model aligns with recent findings by Suhaimi et al.^39^. Thus, TRF’s hypolipidemic properties may contribute to the observed reduction in TNF-α levels. In a hyperlipidemic state, excessive LDL-C undergoes oxidative modification, leading to an increase in oxLDL-C due to heightened oxidative stress. Elevated oxLDL-C levels trigger TNF-α gene expression, further increasing TNF-α levels^26^. In this study, TRF-mediated reduction of LDL-C levels in obese rats subsequently lowered oxLDL-C formation, preventing TNF-α gene activation and thereby reducing vascular TNF-α levels.

Obesity and hyperlipidemia are associated with the progression of atherosclerosis, which is positively correlated with elevated IL-6 levels^45,46^. IL-6 contributes to vascular inflammation by promoting smooth muscle cell proliferation and migration, endothelial dysfunction, and the recruitment and activation of inflammatory mediators, ultimately leading to atherosclerotic plaque formation^45^. In the present study, the administration of TRF in obese rats significantly reduced the pro-inflammatory cytokine IL-6. After six weeks of TRF treatment, IL-6 levels decreased by 42.1% and 45.8% in the OB + TRFHFD and OB + TRFNC groups, respectively, compared to the control OB group. The IL-6 values in obese groups that undergo TRF were comparable to those observed in the NOR + TRFNC group, thus suggesting that TRF normalizes IL-6 levels in obese rats. These findings align with previous research in both animal^47,48^ and human^49,50^ models, supporting the anti-inflammatory effects of TRF and intermittent fasting.

In this study, aortic rings from the OB + TRFHFD and OB + TRFNC groups exhibited thinner vascular IMT compared to the OB group. Foam cells were present in the thoracic aortas of the OB group but absent in the OB + TRFHFD and OB + TRFNC groups. The aortic IMT in the NOR group with TRF was comparable to that of the NOR group. The aortic wall may thicken due to increased collagen deposition and smooth muscle cell proliferation. This process can lead to reduced elasticity of the aorta and impair its ability to regulate blood flow^51,52^. Several factors may contribute to the reduction of aortic IMT in obese rats treated with TRF. First, this effect could be due to increased vascular nitric oxide (NO) production following TRF administration^4^. TRF has been shown to enhance the expression of vascular endothelial nitric oxide synthase, an enzyme responsible for NO production, which increases NO bioavailability. This prevents leukocyte and platelet adhesion to the endothelium, thereby inhibiting vascular smooth muscle cell (VSMC) proliferation and migration^4^. Secondly, TRF’s hypolipidemic effects, observed in this study, may also contribute to reduced aortic IMT. Lower oxLDL-C levels inhibit the production of inflammatory cytokines such as TNF-α and IL-6, preventing foam cell formation and reducing arterial intimal thickening^26,53^. Additionally, the observed decrease in oxidative stress markers after TRF administration may also play a role. TRF’s hypolipidemic effects might reduce the formation of superoxide anions^4,39^, key drivers of VSMC proliferation^54^. Lower superoxide anion levels can prevent the secretion of cyclophilin A (CyPA), a potent monocyte chemoattractant that triggers inflammation^26,55^. Reduced CyPA levels decrease vascular adhesion molecule-1 and E-selectin expression, further limiting VSMC proliferation and intimal thickening^26^.

The histopathological findings of the liver suggest that TRF administration exerts a protective effect on liver tissues in obese rats, particularly those fed with a HFD and normal chow. This effect was supported by significant improvement of the liver enzymes (AST, ALT, and ALP) after TRF administration in obese rats, as seen in this study. This current study and prior reports indicate that obesity, especially when induced by a HFD, often leads to hepatic steatosis characterized by excessive lipid accumulation in hepatocytes, disrupting normal liver architecture^56,57^. The presence of numerous lipid droplets in the OB group confirms this pathological manifestation, which is a hallmark of metabolic dysfunction and a precursor to non-alcoholic fatty liver disease (NAFLD)^58,59^. Interestingly, OB rats subjected to TRF exhibited a notable reduction in lipid accumulation, with liver histology resembling that of normal rats. This suggests that TRF mitigates the detrimental effects of a HFD, likely by improving metabolic homeostasis and enhancing lipid metabolism^60,61^. Prior studies have demonstrated that TRF can regulate circadian rhythms and optimize nutrient utilization, thereby reducing hepatic lipid deposition and improving liver function^62,63^. The absence of significant inflammatory cell infiltration and fibrosis at this magnification further indicates that TRF might prevent the progression of hepatic steatosis into more severe liver pathologies, such as steatohepatitis or fibrosis. This finding is consistent with studies showing that intermittent fasting and TRF modulate inflammatory pathways and oxidative stress in metabolic disorders^64,65^. Overall, the current findings reinforce the potential therapeutic role of TRF in mitigating obesity-induced hepatic damage. By reducing lipid accumulation and preserving liver architecture, TRF may serve as a non-pharmacological intervention for improving liver health in obesity-related metabolic disorders.

Obesity is a well-established risk factor for renal dysfunction, often leading to glomerular hypertrophy, mesangial expansion, and tubulointerstitial fibrosis due to chronic metabolic stress and inflammation^66^. Findings from the current study highlight the detrimental effects of obesity on renal structure and the potential protective role of TRF in mitigating these changes. In the NOR and NOR + TRFNC groups, renal histology revealed well-preserved glomerular structures with no significant signs of damage. This aligns with previous research indicating that normal renal function is maintained in non-obese conditions due to balanced metabolic and hemodynamic regulation^67^. Conversely, the OB group exhibited clear signs of renal injury, including glomerular congestion, tubular injury, and interstitial changes. These pathological alterations are commonly associated with obesity-related kidney disease, where excessive lipid accumulation, systemic inflammation, and oxidative stress contribute to glomerular damage^68^. The observed glomerular hypercellularity and mild shrinkage suggest early glomerulosclerosis, which is a precursor to chronic kidney disease in obesity^69^. Notably, the administration of TRF in obese rats showed substantial improvements in renal histology. The renal tissue largely maintained its normal architecture, and the glomeruli appeared relatively preserved, although some mild mesangial expansion and glomerular capillary wall thickening were observed. These findings suggest that TRF exerts a protective effect on renal tissue, likely by reducing metabolic stress, improving lipid metabolism, and enhancing autophagy^70–72^. These results align with growing evidence that TRF can alleviate metabolic disorders by modulating circadian rhythms and reducing inflammation, both of which play crucial roles in kidney health^73^. Furthermore, TRF has been shown to improve insulin sensitivity and reduce oxidative stress, which may help prevent glomerular injury in obesity^4,39,74^.

The present study has several important limitations that should be acknowledged. One limitation is that the intervention period lasted only six weeks, which may be too short to capture the long-term cardiovascular, hepatic, or renal effects of TRF that typically emerge over prolonged durations. Another important limitation is the relatively small sample size of about six rats per group, which reduces statistical power and limits the generalizability of the findings. In addition, only male rats were studied, and sex-related differences in metabolic and cardiovascular responses may influence the outcomes, thereby limiting applicability across both sexes. The strict TRF protocol applied under controlled laboratory conditions also does not fully reflect the variability and complexity of human eating behaviors in real-world settings. Moreover, the study focused mainly on early biochemical and histological alterations rather than advanced outcomes such as atherosclerotic plaque progression, cardiovascular events, or survival. The high-fat diet followed by a switch to normal chow may likewise not fully replicate the complexity of human dietary habits, and some of the observed benefits could be partly attributable to weight reduction rather than TRF itself, as weight changes were not fully controlled in the analysis. Finally, there were methodological constraints: specialized staining (Oil Red O, Masson’s trichrome, Elastic van Gieson) and immunohistochemical analyses could not be fully performed due to limited tissue samples and insufficient reagents, as many specimens had already been used in previous studies^4^. Consequently, the histological assessment of the thoracic aorta relied mainly on H&E staining, which revealed intima-media thickening, lipid accumulation, and foam cell formation but not hallmark features of advanced atherosclerosis, such as fibrous cap development or extensive elastic lamina disruption. Despite these limitations, the present findings add complementary insights into early structural and biochemical changes, while future studies with larger cohorts, both sexes, longer interventions, and comprehensive histological techniques will be essential to validate and extend these observations.

## Conclusion

Six weeks of TRF improved liver enzyme derangement due to obesity (AST, ALT, and ALP), which was further associated with amelioration of vascular structural changes as well as hepatic and renal tissue morphology. In addition, TRF in HFD-induced obese rats improved vascular oxidative stress and inflammation markers. These beneficial effects were accompanied by reduced TG, LDL-C, and AI, together with an increase in HDL-C.

## Materials and methods

### Preparation of HFD

The experimental rats were provided with two distinct diets: a normal chow (702P Mouse Pellet Feed, Gold Coin, Port Klang, Malaysia) and a specially formulated HFD. The HFD was prepared based on the method outlined by Azemi et al.^4^, consisting of 50% standard chow, 38% ghee, 8% full cream milk, and 4% white sugar. All components were sourced from local markets, and the diet preparation was carried out under clean and hygienic conditions. The nutritional value of both the normal chow and the HFD has been provided in Table 2 below.

**Table 2:** Nutritional value of normal Chow and high-fat diet.

Nutritional value	Normal chow (NC)	High-fat diet (HFD)
Fat **(**g/kg of diet)	30	390
Protein **(**g/kg of diet)	220	61
Carbohydrate **(**g/kg of diet)	580	465
Total ash **(**g/kg of diet)	57	39
Moisture **(**g/kg of diet)	113	45
**Energy (kcal/g)**	∼3.47	∼5.61

### Animals

The experimental animal protocols are approved by the Institutional Animal Care and Use Committee, Universiti Sains Malaysia [USM/IACUC/2020/(126)(1111)] and performed in accordance with the relevant guidelines and regulations, complied with ARRIVE guidelines, and associated guidelines. A total of thirty male Sprague-Dawley rats, aged 12 weeks and weighing between 250 and 300 g, were utilized in this research. The animals were obtained from the Animal Research and Service Centre (ARASC) at the Health Campus of Universiti Sains Malaysia. They were housed in clean polypropylene cages within a temperature-controlled facility, maintained at 25 ± 2 °C with a 12-h light/dark cycle throughout the experimental period.

### Experimental design and sampling

**Fig. 10:**
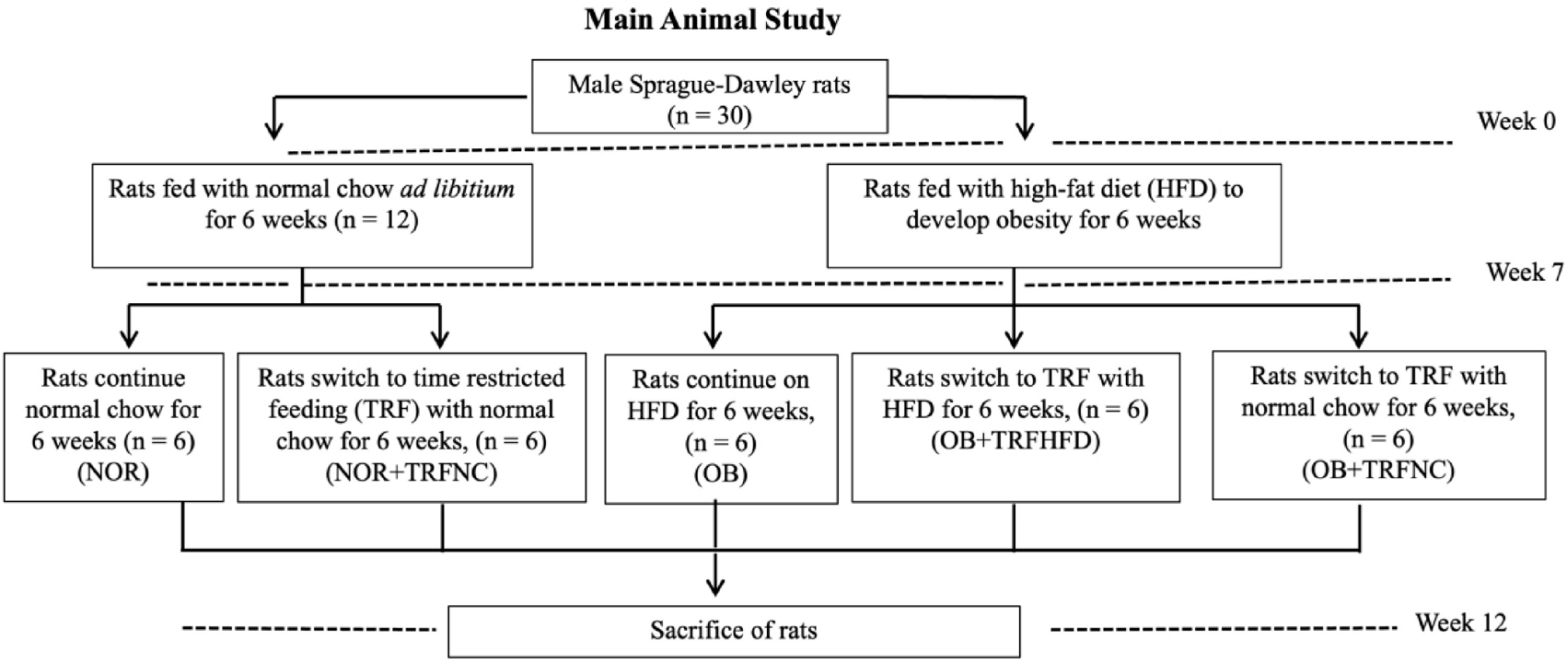
Animal grouping design.

At the outset of the study, the rats were allocated into two primary groups: a normal group and an obese group. The normal group received standard chow, while the obese group was fed a HFD for six weeks to induce obesity. In the seventh week, both normal and obese rats were further subdivided into five experimental groups, with six rats in each group, as depicted in Fig. 10.

Rats assigned to the TRF protocol underwent a daily fasting period of 16 h, from 5:00 p.m. to 9:00 a.m. the following morning. During the remaining 8-h window (9:00 a.m. to 5:00 p.m.), they had unrestricted access to their respective diets. This feeding cycle was maintained for six weeks in the TRF groups. In the twelfth week, all rats were euthanized via intraperitoneal injection of a ketamine (Ketamine 10% Inj, Dutch Farm International BV, Holland) and xylazine (Xylazine 2% Inj, Dutch Farm International BV, Holland) combination (300 mg/kg:30 mg/kg)^26^. Blood samples were collected from the renal arteries, and the serum was subsequently used for biochemical analysis.

### Biochemical analysis of serum

Blood samples were processed by centrifugation at 1,500 × *g* for 25 min using a Kubota 4000 centrifuge (Japan). The separated serum was subsequently analyzed to determine lipid profile parameters. Concentrations of TC, TG, and HDL-C were quantified using a colorimetric assay with the Integra 800 automated immunoanalyzer (Roche Diagnostics, Mannheim, Germany). LDL-C levels were estimated using the Friedewald Equation^26^ as follows:

$${\text{LDL}} - {\text{C }} = {\text{ TC }} - \left( {{\text{HDL}} - {\text{C}} + {\text{TG}}/5} \right)$$

The AI is a strong marker for predicting the risk of atherosclerosis and coronary heart disease^75^. Its formula is based on LDL-C and HDL-C values in the serum^26^, calculated as follows:

$${\text{AI}} = {\text{LDL}} - {\text{C}}/{\text{HDL}} - {\text{C}}$$

The liver function test was measured according to the method described by Zakaria et al.^76^. The collected serum was subjected to the determination of AST, ALT, and ALP levels using the Integra 800 automatic immunoanalyzer (Roche Diagnostic Systems, Mannheim, Germany)^76^.

### Biochemical analysis of aortic tissue

Thoracic aorta tissues were carefully excised from the rats (50 µg) and homogenized in 500 µL radioimmunoprecipitation assay (RIPA) buffer (20–188, Sigma-Aldrich, St. Louis, MO, USA) supplemented with 0.05% protease inhibitor cocktail (Sigma-Aldrich) (1:10 w/v)^77^. The homogenates were then centrifuged at 3,000 × g for 20 min at 4 °C, and the resulting supernatants were collected for analysis. Total protein content was quantified using a commercial protein assay kit (704002, Cayman Chemical, Michigan 48108, USA), and the supernatants were subsequently analyzed for SOD, MDA, TNF-α, and IL-6. Aortic SOD (Catalog No.: QY-E11476) and MDA (Catalog No.: QY-E11488) levels were measured using enzyme-linked immunosorbent assay (ELISA) kits obtained from Qayee Bio-Technology Co., Ltd (Shanghai, China). Similarly, aortic TNF-α and IL-6 levels were determined using ELISA kits (TNF-α, Catalog No.: QY-E10880; IL-6, Catalog No.: QY-E11509) according to the manufacturer’s instructions (Qayee Bio-Technology Co., Ltd, Shanghai, China).

### Histology of thoracic aorta, liver, and renal tissues

Histological assessments were performed based on the protocols adapted from Azemi et al. and Zakaria et al.^26,76^. Samples from the thoracic aorta, liver, and kidney were fixed in 10% neutral buffered formalin (SF98-4, Fischer Scientific, Selangor, Malaysia). Following fixation, the tissues were dehydrated through a graded ethanol (LSMETOH-ABS, Fisher Scientific, Selangor, Malaysia) series using a tissue processor (TP 1020, Leica, Solms, Germany), embedded in paraffin (39601006, Leica Paraplast, Leica Biosystems, IL 60010, USA), and sectioned into 4 μm slices. These sections were mounted on glass slides (7101, Hawach Scientific, Shaanxi Province, China) and stained with hematoxylin (3801562, Leica Biosystems, IL 60010, USA) and eosin (8801600, Leica Biosystems, IL 60010, USA) (H&E). Microscopic examination was conducted at 100 to 200× magnification using a light microscope (Olympus BX41, Olympus America Inc., Center Valley, PA, USA), and images were analyzed with the cellSens software (Olympus America Inc.). For thoracic aorta analysis, four radial positions of each tissue Sect. (0°, 90°, 180°, and 270°) were selected to assess IMT. Additional evaluations included observations of vascular wall integrity, structural disruptions, and foam cell presence. In liver tissue, histological analysis focused on the degree of hepatic steatosis, including lipid droplet accumulation and overall tissue architecture. The liver lesion scoring was evaluated according to the methods described by Schneider et al.^78^ and Wong et al.^79^. Kidney sections were examined for early structural damage, particularly changes in the glomeruli and glomerular capillary walls.

### Statistical analysis

Results were presented as mean ± standard error of the mean (SEM). One-way analysis of variance (ANOVA) followed by a Tukey *post-hoc* test was conducted for multiple comparison testing. Tests were conducted and graphs were prepared using GraphPad Prism software version 8.

## Data Availability

All the data generated or analyzed in the current study are included within this article.
